# Eicosapentaenoic Acid Activates RAS/ERK/C/EBPβ Pathway through H-Ras Intron 1 CpG Island Demethylation in U937 Leukemia Cells

**DOI:** 10.1371/journal.pone.0085025

**Published:** 2014-01-13

**Authors:** Veronica Ceccarelli, Giuseppe Nocentini, Monia Billi, Serena Racanicchi, Carlo Riccardi, Rita Roberti, Francesco Grignani, Luciano Binaglia, Alba Vecchini

**Affiliations:** 1 Department of Experimental Medicine, University of Perugia, Perugia, Italy; 2 Department of Medicine, University of Perugia, Perugia, Italy; INRA, France

## Abstract

Epigenetic alterations, including aberrant DNA methylation, contribute to tumor development and progression. Silencing of tumor suppressor genes may be ascribed to promoter DNA hypermethylation, a reversible phenomenon intensely investigated as potential therapeutic target. Previously, we demonstrated that eicosapentaenoic acid (EPA) exhibits a DNA demethylating action that promotes the re-expression of the tumor suppressor gene CCAAT/enhancer-binding protein δ (C/EBPδ). The C/EBPβ/C/EBPδ heterodimer formed appears essential for the monocyte differentiation commitment. The present study aims to evaluate the effect of EPA on RAS/extracellular signal regulated kinases (ERK1/2)/C/EBPβ pathway, known to be induced during the monocyte differentiation program. We found that EPA conditioning of U937 leukemia cells activated RAS/ERK/C/EBPβ pathway, increasing the C/EBPβ and ERK1/2 active phosphorylated forms. Transcriptional induction of the upstream activator H-Ras gene resulted in increased expression of H-Ras protein in the active pool of non raft membrane fraction. H-Ras gene analysis identified an hypermethylated CpG island in intron 1 that can affect the DNA-protein interaction modifying RNA polymerase II (RNAPII) activity. EPA treatment demethylated almost completely this CpG island, which was associated with an enrichment of active RNAPII. The increased binding of the H-Ras transcriptional regulator p53 to its consensus sequence within the intronic CpG island further confirmed the effect of EPA as demethylating agent. Our results provide the first evidence that an endogenous polyunsaturated fatty acid (PUFA) promotes a DNA demethylation process responsible for the activation of RAS/ERK/C/EBPβ pathway during the monocyte differentiation commitment. The new role of EPA as demethylating agent paves the way for studying PUFA action when aberrant DNA methylation is involved.

## Introduction

In addition to genetic aberrations, epigenetic changes play a major role as an alternative mechanism for transcriptional inactivation of cancer-related genes [1], [2]. Intensely studied is the DNA methylation process, an epigenetic modification that occurs on the cytosine in CpG dinucleotides, essential for gene silencing in cancer cells [3]. All cancer types possess aberrant DNA methylation, characterized by global genomic hypomethylation and, yet at the same time, localized hypermethylation of “CpG islands”, within the promoter region of tumor suppressor genes [4]. Many genes regulating critical cellular pathways may be targeted for aberrant CpG islands methylation in all forms of neoplasia [4]. DNA hypermethylation is associated with a closed chromatin structure which induces transcriptional silencing of the associated genes, but, in contrast to genetic aberrations, it is a reversible phenomenon. As a consequence, changes on DNA methylation levels can modify gene expression [5]. The development of human epigenomic projects [6] and epigenetic therapies [7], [8] is a clear demonstration of how epigenetic changes can modify gene expression. Demethylating strategies contribute to re-express DNA-methylated tumor suppressor genes in cancer cells [9].

Recently we found that in U937 leukemia cells eicosapentaenoic acid (EPA), a newly-synthesized or dietary polyunsaturated fatty acid (PUFA), induces the expression of tumor suppressor gene CCAAT/enhancer-binding protein δ (C/EBPδ) by a site-specific CpG promoter demethylation [10]. In addition, EPA enhances the expression of C/EBPβ, a key transcription factor in monocyte differentiation program [11], and promotes C/EBPβ/C/EBPδ heterodimer formation, inducing the expression of macrophage colony-stimulating factor (M-CSF) receptor [10], an early gene specific for the monocyte/macrophage cell lineage differentiation process [12]. The effects of EPA observed are concordant with the claimed anticarcinogenic effect of PUFA and prompt the research on the molecular and cellular mechanisms, which remain still relatively unknown [13].

It can be speculated that an active C/EBPβ form is responsible for the binding of C/EBPβ/C/EBPδ heterodimer to M-CSF receptor promoter. Phosphorylation on Thr235 is essential for C/EBPβ activation by the oncogenic Ras proteins through extracellular-signal-regulated kinase (ERK) pathway [14]. Indeed, C/EBPβ Thr235 represents an ERK1/2 phosphorylation site that is essential to promote the ability of C/EBPβ to bind DNA and induce transcription of target genes [14]. Activation of this pathway was observed in leukemia cells undergoing the monocyte differentiation process [15]. Moreover, endogenous levels of ERK1/2 are active when exhibiting phosphorylated forms [16]. Signaling through activated ERK1/2, an essential step in the differentiation of myeloid cells along the monocyte/macrophage lineage [17], occurs via Ras pathway [18]. Consistent with these data is the induction of monocyte differentiation by H-Ras activation [19]. Indeed, when activated H-Ras was highly expressed in the U937 cell line, monocyte differentiation was observed [20]. Ras proteins transmit signals from key fate-determining cytokine receptors, such as M-CSF receptor [21], whose expression levels are induced in U937 leukemia cells after EPA treatment [10].

In the present study, to unravel the molecular mechanisms through which EPA promotes monocyte differentiation commitment in U937 promonocytic cell line, we evaluated the effect of EPA treatment on the Ras/ERK/C/EBPβ pathway. We found that C/EBPβ and ERK1/2 proteins exhibit active phosphorylated forms after PUFA conditioning. Moreover, EPA induces H-Ras isoform mRNA and protein expression through demethylation of a CpG island in intron 1. This is the first demonstration that a DNA demethylation process induced by EPA is responsible for the activation of Ras/ERK/C/EBPβ pathway.

## Materials and Methods

In all the experiments inhibition of cell cycle progression and changes on cellular morphology induced by PUFA in U937 leukemia cells were confirmed [10].

### Materials

Oleic acid (18∶1, n-9; OA), linoleic acid (18∶2, n-6; LA), α-linolenic acid (18∶3, n-3; LNA), arachidonic acid (20∶4, n-6; AA), eicosapentenoic acid (20∶5, n-3; EPA), docosahexaenoic acid (22∶6, n-3; DHA), bovine serum albumin fraction V (BSA, fatty acid free), and 5-aza-2′-deoxy-cytidine (5-aza-dC) were from Sigma.

### Preparation of albumin-bound fatty acid

A stock solution of each fatty acid (5 or 10 mM) was prepared as previously described [10].

### Cell culture and treatments

U937 promonocytic human cell line (CRL-1593.2) was obtained from the American Type Culture Collection and cultured as in our previous work [10]. Cells were seeded at a density of 0.3×10^6^ cells per ml for all experiments. Cells were incubated with fatty acid/BSA solutions (100 µM final concentration) at the indicated times.

### Isolation and analysis of caveolae-raft enriched membrane fraction

Caveolae-raft membrane fractions were isolated according to an established method with the following modifications [22], [23]. Briefly, 200×10^6^ untreated and fatty acid treated U937 cells (24 hours, 100 µM) were washed with PBS and lysed with 0.8 ml of MES-buffered saline (25 mM 2-(N-Morpholino) ethanesulfonic acid 4-Morpholineethane sulfonic acid (MES) (pH 6.5), 0.15 M NaCl containing 1% (v/v) Triton X-100 and protease inhibitors (1 mM NaVO_4_, 1 µg/ml aprotinin, 2 µg/ml leupeptin, and 1 mM PMSF). Cells were homogenized and centrifuged at 2000×g to remove nuclei and large cellular debris. All steps were done at 0–4°C. The supernatant was adjusted to 40% sucrose and the solution (1.6 ml) was placed at the bottom of an ultracentrifuge tube and overlaid with 1.75 ml of 30% sucrose solution and 1.75 ml of 5% sucrose solution containing 25 mM MES (pH 6.5) and 0.15 M NaCl. The discontinuous sucrose gradient was centrifuged for 16 hours in SW50.1 rotor at 200.000×g at 4°C (Bekman Instrument). After centrifugation 12×0.425 ml sucrose gradient fractions were collected manually from the top of the gradient. A white light-scattering band corresponding to fractions 4 and 5 (Triton insoluble protein) represented the caveolae-raft enriched membrane fraction (named raft membrane fraction). Fractions 9–12 (Triton soluble protein), containing about 99% of the total cellular membrane proteins, were pooled (named non raft membrane fraction). Raft and non raft fractions were maintained at −20°C for further analyses.

Raft proteins content was quantified according to Bradford using bovine serum albumin as standard [24]. Lipids were extracted according to Folch [25] and stored in benzene at −20°C under nitrogen. Total phospholipids were quantitated as inorganic phosphate according to Itoh [26]. Phospholipid classes were isolated by two dimensional thin layer chromatography on silica gel G. Cholesterol was separated by thin-layer chromatography (n-hexane/diethyl ether/acetic acid; 70∶30∶1, v/v/v/). Lipids were visualized with Cu-acetate reagent [27] and images were acquired using the VersaDoc Imaging System (Bio-Rad). Signals were quantified using Quantity One software (Bio-Rad) by referring to the concentration of authentic lipid standards.

The fatty acid content of raft and non raft membranes was evaluated by gas chromatographic analysis of the fatty acid methyl esters obtained by transmethylation of the extracted lipids. A Shimadzu GC-14A gas chromatograph equipped with a flame ionization detector (250°C) and a fused silica capillary column Supercowax™10 (30 m–0.32 mm, internal diameter) was used. Individual fatty acid methyl esters were identified by referring to authentic standards.

### qRT-PCR

Total RNA was extracted from control and fatty acid treated (1–24 hours, 100 µM) U937 cells using the TRIzol reagent (Invitrogen), according to the manufacturer's guidelines. Reverse transcription was performed using Quanti Tect Reverse Transcription Kit (Qiagen). qRT-PCR was performed with a Chromo 4 (MJ Research Bio-Rad) real time cycler using the specific FAM/MGB dye-labelled TaqMan probes: H-Ras (Hs00978051_g1), N-Ras (Hs00180035_m1), and K-Ras (Hs00364284_g1). Gene expression was quantified relative to the expression of endogenous control human hypoxanthine-guanine phosphoribosyl transferase (HPRT). VIC/MGB probe was amplified in the same tube of investigated genes. Probes were purchased from Applied Biosystem. All experiments were carried out in triplicate and the ΔΔ^Ct^ method was used to determine expression of the genes of interest, as previously described [28].

Quantitative gene expression analysis for H-Ras exon 1 and exon 2 were performed using Mx3000P™ Real-Time PCR System with Brilliant® SYBR® Green QPCR Master Mix (Stratagene) and ROX as reference dye. Quantitative PCR reactions were performed under conditions standardized for each primer set. Each experimental time was investigated with four replicates of three independent treatments. The primers used were the followings. H-Ras exon1: *for*, 5′-TGCCCTGCGCCCGCAACCCGAG-3′; *rev*, 5′-CGTTCACAGGCGCGACTGCC-3′. H-Ras exon 2: *for*, 5′CAGGAGACCCTGTAGGAGGA-3′; *rev*, 5′-GGATCAGCTGGATGGTCAGC-3′. Human HPRT mRNA was the house-keeping gene. The ΔΔ^Ct^ method was used to determine modulation of the mRNA level of each exon [28].

### Immunoblot analysis

U937 cells were cultured in standard conditions with fatty acids (24 hours, 100 µM) or 5-aza-dC (daily additions for 2 days, 1 µM f.c.). Protein samples from total cell lysates (50 µg) were subjected to SDS-polyacrilamide gel electrophoresis, electroblotted onto a nitrocellulose membrane (Schleicher and Schuell), and probed using the following antibodies: anti-phospho-C/EBPβ (Thr235) #3084, anti-phospho-p44/42 MAPK (pERK1/2, T-202/Y-204) #9101, anti-p44/42 MAPK #9102, anti-pan-Ras #3965 (Cell Signaling). Anti-H-Ras specific antibody 18295-1-AP (Proteintech), anti-N-Ras (F155:sc-31) and anti-K-Ras (F234:sc-30) (Santa Cruz Biotechnology). Immunoreactive bands were visualized using the ECL assay (Amersham Pharmacia Biotech, Amersham). Anti-β-tubulin antibody (Sigma-Aldrich) was used to normalize. Images were acquired using the VersaDoc Imaging System (Bio-Rad), and signals were quantified using Quantity One software (Bio-Rad).

### DNA isolation and quantitative DNA methylation analysis of C/EBPβ and H-Ras CpG islands

Genomic DNA from control U937 cells or U937 grown for 24 hours with 100 µM OA or 100 µM EPA was extracted using FlexiGene DNA Kit (Qiagen). EMBOSS (European Molecular Biology Open Software Suite) and MethPrimer on-line software programs (University of California San Diego web site) were used to identify potential CpG islands for C/EBPβ, N-Ras, and H-Ras genes. DNA methylation levels were quantified for human C/EBPβ (MePH25981-3A) and H-Ras (MePH14574-1A) (Qiagen) using Methyl-Profiler qPCR Primer Assay. qRT-PCR program was performed as indicated in the manual instructions. Analysis of DNA methylation status of CpG islands was carried out using restriction enzyme digestion (DNA Methylation Enzyme kit MeA-03, Qiagen) followed by SYBR Green-based real time PCR detection as previously described [10]. The relative amount of each DNA fraction (methylated and unmethylated) was calculated using ΔCt method [10].

### Bisulfite modification of genomic DNA and sequencing

Genomic DNA was obtained from U937 cells, control or grown for 24 h with 100 µM OA or 100 µM EPA, using FlexiGene DNA Kit (Qiagen). The bisulfite reaction to determine DNA methylation status was performed as previously described [29], [30]. The DNA fragments covering N-Ras CpG island (−29/+171) and H-Ras CpG island B (640/882) were amplified by PCR using the following primers. N-Ras: *for*, 5′-AAAGTTTTATTGATTTTTGAGATATTAGTA-3′; *rev*, 5′-TTTAAACAAATTTAAAACCACAACC-3′. H-Ras: *for*, 5′-AGTTTTTTGTGGTTGAAAGATGTT-3′; *rev*, 5′-ACACCCAAATTAAAAACTACTAAATC -3′. The PCR products were cloned into pCR2.1 TOPO (Invitrogen) and six clones randomly picked from each of two independent PCRs were sequenced using T7 primer at the Genechron-Ylichron Laboratory (Rome).

### Chromatin immunoprecipitation

ChIP assays were performed on U937 cells (about 10^6^), control or grown with 100 µM OA or EPA for 24 hours, using the EZ-Chip kit (Millipore-Upstate). Cells were cross-linked and cell lysates sonicated until chromatin fragments became 200–1.000 bp in size. Mouse RNAPII 8WG16 monoclonal antibody MMS-126R (Covance) or rabbit p53 antibody #9282 (Cell Signaling) were used for immunoprecipitation. Mouse or rabbit IgG (Millipore) were used as a negative control. After immunoprecipitation, recovered chromatin samples were subject to qRT-PCR with Brilliant SYBR Green qPCR Master Mix. In RNAPII assays the H-Ras sequences amplified were within i) exon 1 (1/+135), ii) intron 1 region C (+136/+639), iii) CpG island B (+640/+882), iv) intron 1 region D (+883/+1167), and v) exon 2 (+1168/+1331). The following primers were used. i) Exon 1: *for*, 5′-TGCCCTGCGCCCGCAACCCGAG-3′; *rev*, 5′-CGTTCACAGGCGCGACTGCC-3′. ii) Intron 1 region C: *for*, 5′-GTGAACGGTGAGTGCGGGCA-3′; *rev*, 5′-CGCGCCGCGCGTATTGCTGC-3′. iii) CpG island B: *for*, 5′-CCTGTTCTGGAGGACGGTAA-3′; *rev*, 5′-GTCGGCAGAAAGGCTAAAGG-3′. iv) Intron 1 region D: *for*, 5′-TCAGATGGCCCTGCCAGCAG-3′; *rev*, 5′-TCCTCCTACAGGGTCTCCTG-3′. v) Exon 2: *for*, 5′CAGGAGACCCTGTAGGAGGA-3′; *rev*, 5′-GGATCAGCTGGATGGTCAGC-3′. In p53 assays the sequence containing the p53 element of CpG island B was amplified using the following primers: *for*, 5′-CGCTCAGCAAATACTTGTCGG-3′; *rev*, 5′-TTACCGTCCTCCAGAACAGG-3′. Data were analyzed quantitatively according to the formula 2^−Δ[*C*(IP)−*C*(input)]^−2^−Δ[*C*(control IgG)−*C*(input)]^ [31].

PCR was performed using AmpliTaq Gold (Applied Biosystems-Roche) and the above p53 primers. Cycling conditions were: 10 min at 95°C, 30 s at 95°C (35 cycles), 30 s at 60°C, and 30 s at 72°C.

### Statistical analyses

All the results are presented as mean ± S.D and were analyzed by one-way ANOVA with Bonferroni's post test. A p-value of less than 0.05 was considered significant.

## Results

### PUFA induce C/EBPβ and ERK1/2 phosphorylation, and increase Ras proteins expression

**Figure 1:**
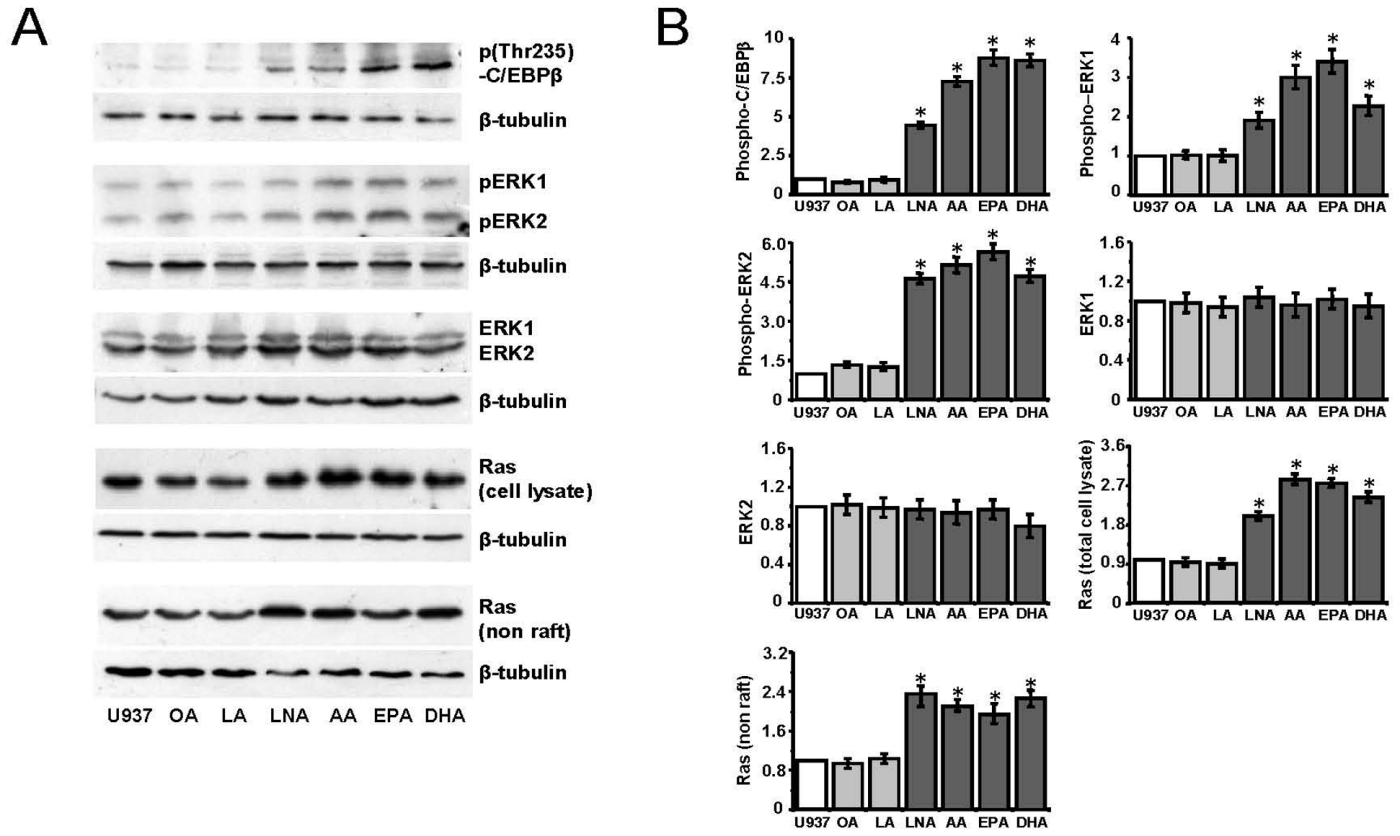
Effect of fatty acids on Ras, ERK1/2, and phospho-C/EBPβ protein levels. (A) U937 cells were treated with 100 µM fatty acids (OA, oleic; LA, linoleic; LNA, α-linolenic; AA, arachidonic; EPA, eicosapentaenoic; DHA, docosahexaenoic) for 24 hours. Total cell lysates or isolated non raft membrane fractions (50 µg protein) were subjected to Western blotting with the indicated antibodies. Pan-Ras Ab was used to detected all Ras isoforms. For each protein, one representative out of three experiments is reported. (B) Quantitative analysis. The chart shows normalized Western blot band densities, presented as fold induction with respect to U937 control cells. Images of independent blots were acquired using the Versadoc Imaging System and signals were quantified using Quantity One Software. Data are the means ± S.D. of three independent experiments. (*, p<0.05)

Phosphorylation of C/EBPβ at Thr235 is essential for its transcriptional ability [14]. We measured the levels of pC/EBPβ in control U937 cells and after fatty acid conditioning. C/EBPβ phosphorylated form was evident in LNA, AA, EPA, and DHA treated cells, whereas it was barely detectable in control, and OA or LA treated U937 (Fig. 1). Activated C/EBPβ induces its own gene transcription [11], which could be responsible for the increase of C/EBPβ expression during monocyte differentiation commitment [10]. We performed C/EBPβ gene analysis and found a putative CpG island (−794/+478) that was completely unmethylated in control U937 cells, indicating that C/EBPβ promoter may be accessible to phosphorylated C/EBPβ [10].

We examined whether the observed C/EBPβ post-transcriptional changes derive from the activation of the Ras/ERK pathway [15]. Similar to C/EBPβ, ERK1/2 exhibited active phosphorylated forms upon PUFA treatment (Fig. 1A and B), consistent with C/EBPβ Thr235 as an ERK phosphorylation site [14]. On the contrary, ERK1/2 proteins expression was unchanged (Fig. 1A and B). Interestingly, ERK pathway activation was accompanied by the increase of total Ras proteins expression (Fig. 1A and B). None of the proteins was affected by OA and LA treatment (Fig. 1A and B).

### Fatty acid treatment does not affect raft lipid composition

Ras proteins may be localized in raft and non raft cellular membranes. Activated Ras protein isoforms reside predominantly in the non raft membrane fraction [32], [33]. PUFA modify the structure and composition of membrane rafts, thus affecting membrane-associated signaling proteins such as Ras [34], [35]. For this reason we verified the Ras proteins membrane localization in U937 leukemia cells exhibiting ERK/C/EBPβ pathway activation after PUFA treatment. We found that the Ras proteins were localized in non raft membranes and were increased in this fraction after PUFA conditioning (Fig. 1A and B). No immunoblot signal for Ras proteins was detected in the raft membrane fraction of control and fatty acids treated U937 cells.

Proteins, total phospholipids, phospholipid classes, and cholesterol levels were unchanged in raft membranes in any of the studied conditions. The analysis of total fatty acid composition showed an increase of each added fatty acid in the non raft membranes (Table 1), whereas no differences were found in the raft fraction (Table 2), suggesting that fatty acids were unable to enter and modify the raft membrane structure.

**Table 1:** Fatty acid composition of total lipids from non raft membranes fraction.

	U937	OA	LA	LNA	AA	EPA	DHA
16∶0	36.3±2.1	29.1±3.6	27.2±2.3	32.5±4.1	36.2±2.9	32.0±3.3	34.6±4.1
16∶1 (n-9)	1.6±0.3	1.4±0.4	0.6±0.2	0.4±0.1	1.1±0.2	0.9±0.1	0.5±0.1
18∶0	13.4±2.9	7.7±1.2	14.0±1.6	18.4±2.1	17.9±0.9	20.5±2.5	16.3±2.3
18∶1 (n-9)	33.2±3.2	53.7±4.5	18.2±2.1	17.9±1.8	14.0±1.9	11.4±1.2	30.2±3.2
18∶2 (n-6)	3.1±0.5	1.6±0.4	28.1±2.9	2.6±0.3	1.9±0.1	1.6±0.3	2.9±0.7
18∶3 (n-3)	0.8±0.2	0.2±0.1	0.3±0.1	21.2±2.5	0.2±0.1	0.3±0.1	0.2±0.1
20∶2 (n-6)	0.3±0.1	0.2±0.1	2.8±0.4	0.3±0.1	0.3±0.1	0.2±0.1	0.3±0.1
20∶3 (n-3)	0.7±0.1	0.4±0.1	1.4±0.3	0.2±0.1	0.4±0.1	0.4±0.1	1.1±0.3
20∶4 (n-6)	6.1±0.8	3.0±0.5	4.2±0.4	3.1±0.4	21.0±2.9	2.0±0.4	5.6±0.3
20∶5 (n-3)	0.5±0.1	0.4±0.1	0.3±0.1	0.8±0.2	0.3±0.1	20.3±1.8	1.6±0.2
22∶4 (n-6)	0.6±0.1	0.3±0.1	0.4±0.1	0.4±0.1	5.4±0.9	0.4±0.1	0.4±0.1
22∶5 (n-3)	1.3±0.2	0.8±0.1	1.0±0.1	1.3±0.2	0.4±0.1	8.8±1.5	0.8±0.1
22∶6 (n-3)	2.1±0.4	1.2±0.3	1.5±0.1	0.9±0.1	0.9±0.1	1.2±0.1	5.5±0.9

**Table 2:** Fatty acid composition of total lipids from raft membranes fraction.

	U937	OA	LA	LNA	AA	EPA	DHA
16∶0	51.6±3.2	53.6±4.1	52.7±2.9	50.0±3.3	50.5±2.7	52.3±1.9	55.6±4.1
16∶1 (n-9)	1.7±0.2	1.4±0.2	1.7±0.3	1.2±0.2	0.9±0.1	0.9±0.1	1.8±0.3
18∶0	42.0±2.2	41.2±1.9	39.7±3.5	43.2±4.6	41.4±2.7	42.4±2.9	38.8±4.0
18∶1 (n-9)	1.5±0.2	1.1±0.2	3.0±0.1	1.1±0.1	2.1±0.3	1.3±0.2	1.3±0.1
18∶2 (n-6)	0.4±0.1	0.4±0.1	0.3±0.1	1.0±0.1	0.8±0.1	0.3±0.1	0.2±0.1
18∶3 (n-3)	0.12±0.06	0.13±0.03	0.20±0.02	0.46±0.06	0.62±0.03	0.13±0.01	0.11±0.02
20∶2 (n-6)	0.43±0.08	0.62±0.05	0.44±0.03	0.33±0.01	0.64±0.07	0.81±0.1	0.72±0.12
20∶4 (n-6)	0.7±0.1	0.8±0.2	0.8±0.1	1.2±0.1	1.1±0.2	0.8±0.1	0.6±0.1
22∶4 (n-6)	0.41±0.1	0.25±0.1	0.80±0.1	0.72±0.09	0.75±0.08	0.51±0.04	0.45±0.06
22∶6 (n-3)	1.10±0.03	0.51±0.01	0.43±0.06	0.81±0.06	1.23±0.09	0.61±0.05	0.43±0.03

### Effect of EPA on Ras isoforms expression

**Figure 2:**
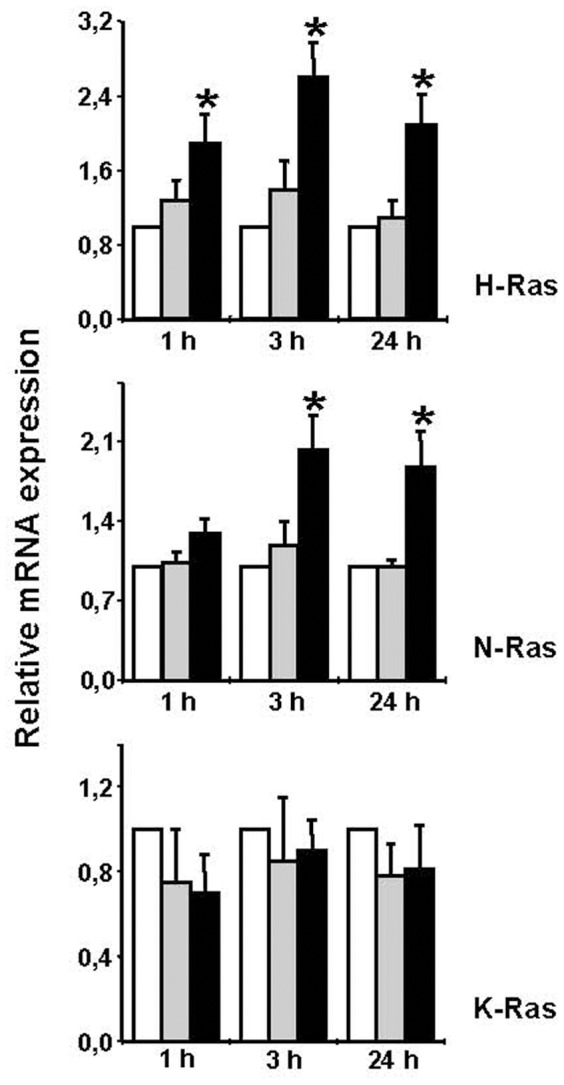
Effect of EPA on Ras isoforms expression. mRNA content was evaluated for H-Ras, N-Ras, and K-Ras after 1-, 3-, and 24-h treatment with 100 µM fatty acids, using qRT-PCR. *White bars*, control U937; *gray bars*, OA; *black bars*, EPA. Data are presented as relative expression by calculating 2^−ΔΔCt^ normalized to untreated U937 cells. The means ± S.D. of three separate experiments are shown (*, p<0.01).

To investigate whether the increased Ras proteins expression induced by PUFA is imputable to transcriptional events, qRT-PCR was performed for H-Ras, N-Ras and K-Ras isoforms. The expression kinetic profile was evaluated by mRNA level in control U937 cells and after 1, 3, and 24 hours treatment with OA or EPA, as potential inactive and active inducers, respectively. A significant increase of H-Ras and N-Ras mRNA levels was observed after EPA conditioning for 1 and 3 hours, respectively (Fig. 2). On the contrary, OA did not induce significant changes at any of the times studied. K-Ras mRNA levels were not affected by EPA and OA (Fig. 2), indicating that this isoform is not involved in the increase of Ras proteins expression.

**Figure 3:**
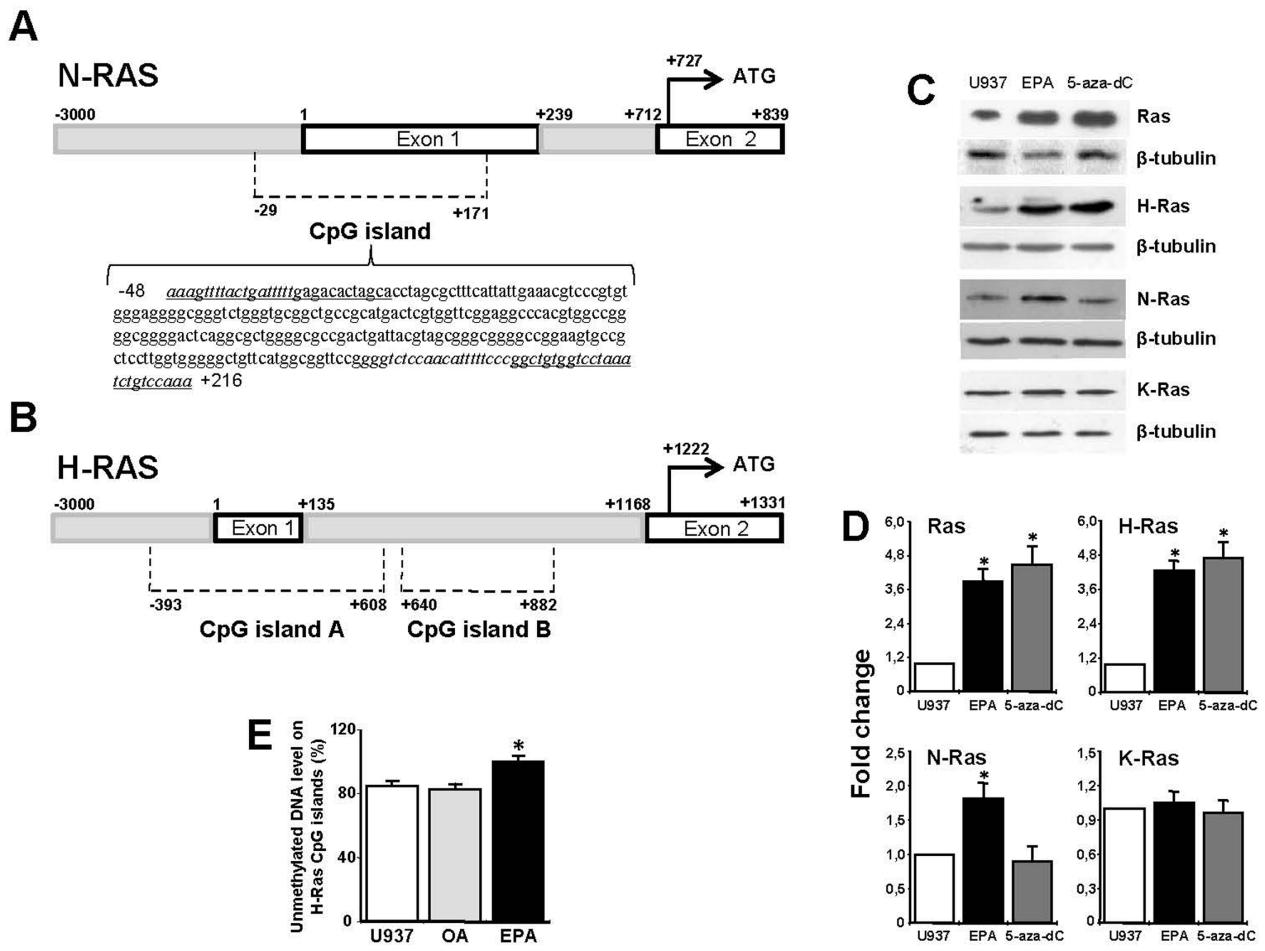
CpG island analysis of Ras gene isoforms and effect of EPA and 5-aza-dC on Ras protein expression in U937 cells. (A) N-Ras gene (−3000/+839) analysis and nucleotide sequence of the CpG island. The underlined sequences indicate the forward and reverse primers utilized for cloning and sequencing after bisulfite reaction. The nucleotide sequences flanking the CpG island are in italics. (B) H-Ras gene (−3000/+1331) CpG islands. (C) Levels of Ras protein isoforms after 100 µM EPA (24 h) or 1 µM 5-aza-dC (2 days). Total cell lysates (50 µg protein) were subjected to Western blotting with the indicated antibodies. Ras was detected with pan-Ras Ab. For each protein, one representative out of three experiments is reported. (D) Quantitative analysis. The chart shows normalized Western blot band densities, presented as fold induction with respect to U937 control cells. Images of independent blots were acquired using Versadoc Imaging System and signals were quantified using Quantity One Software. Data are the means ± S.D. of three independent experiments. (*, p<0.01). (E) Methylation status of H-Ras gene CpG islands. U937 cells were treated with 100 µM OA or EPA for 24 h and the percent of unmethylated DNA of H-Ras CpG islands was quantified using the Methyl-Profiler qPCR Primer Assay. The means ± S.D. of three separate experiments are shown (*, p<0.001 versus OA-treated or U937 untreated cells).

To understand the molecular mechanism by which EPA increased H-Ras and N-Ras mRNA levels, we hypothesized a DNA demethylation process. Indeed, EMBOSS (European Molecular Biology Open Software Suite) and MethPrimer online software programs identified potential methylated CpG islands. The analyzed sequences for both H-Ras and N-Ras genes contained 3000 bp upstream the transcription start site, untranslated exon 1, intron 1, and exon 2, which contains the translation start ATG (Fig. 3A and B). A 200-bp CpG island (−29/+171) containing 22 CpG dinucleotides was found in N-Ras sequence (Fig. 3A). The analysis of H-Ras gene (−3000/+1331) retrieved two CpG islands, henceforward termed islands A and B (Fig. 3B). Island A (1001 bp, −393/+608) spans the proximal promoter region, untranslated exon 1, and a 5′ portion of intron 1. Island B (242 bp, +640/+882) is located in an internal region of intron 1, downstream CpG island A and 286 bp upstream the start site of exon 2 (Fig. 3B).

The hypothesis that a demethylation process could be involved in the increase of Ras proteins expression was verified in U937 cells treated with the DNA demethylating agent 5-aza-dC (2 days, 1 µM) and EPA (24 hours, 100 µM). Either treatment produced an increase of total Ras and H-Ras proteins expression, supporting the involvement of a demethylation process. N-Ras was induced only after EPA conditioning and K-Ras was not affected by any treatment (Fig. 3C and D). We performed bisulfite sequencing of the N-Ras CpG island in untreated U937 cells. All CpG were unmethylated (Fig. 3A), justifying the lack of induction after 5-aza-dC (Fig. 3C and D). Factors other than DNA demethylation were responsible for the increased N-Ras expression after EPA treatment.

We next quantified the DNA methylation levels of H-Ras CpG islands by measuring the percent content of unmethylated DNA by Methyl-Profiler qPCR Primer Assay in untreated U937 cells and after OA or EPA. Quantitative RT-PCR indicated that about 85% of H-Ras DNA copies were unmethylated in control U937 cells. This value increased to about 100% (p<0.001) after EPA conditioning (Fig. 3E). Although only a small portion (about 15%) of DNA CpG dinucleotides present in the two islands was methylated, it appears to be essential for the increase of H-Ras gene expression, as confirmed by the effect of the demethylating agent 5-aza-dC (Fig. 3C and D)

### EPA enhances H-Ras exon 2 transcription level

**Figure 4:**
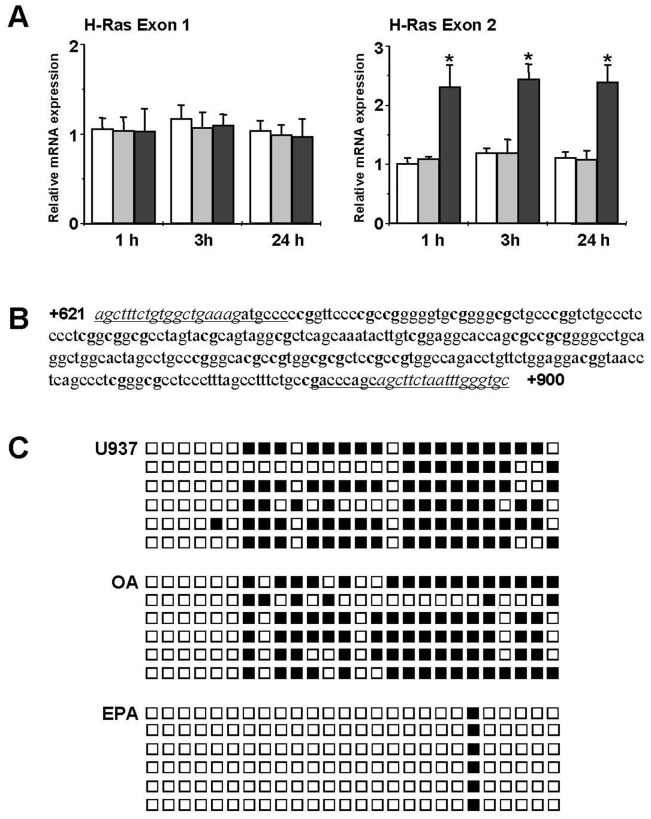
EPA increases H-Ras exon 2 transcription and demethylates intron 1 CpG island B. (A) mRNA content was evaluated for H-Ras exon 1 and exon 2 after 1-,3-, and 24-h treatment with 100 µM fatty acids using qRT-PCR. *White bars*, control U937 cells; *gray bars*, OA; *black bars*, EPA. Data are presented as relative expression by calculating 2^−ΔΔCt^ normalized to untreated U937 cells. The means ± S.D. of three separate experiments are shown (*, p<0.01). (B) H-Ras intron 1 CpG island B sequence. The underlined sequences indicate the forward and reverse primers utilized for cloning and sequencing after bisulfite reaction. The cloned fragment (242 bp) contains 26 CpGs (in bold). The nucleotide sequences outside the CpG island B are in italics. (C) Sequencing of individual clones generated by PCR after bisulfite reaction. Black and white square represent methylated and unmethylated CpGs, respectively.

To localize the methylated cytosines on CpG islands A and B (Fig. 3B) we measured the mRNA levels of H-Ras exons 1 and 2. Quantitative RT-PCR amplification of exon 1 exhibited no differences among U937 control cells and OA or EPA conditioning up to 24 hours (Fig. 4A), suggesting that the H-Ras promoter region located in CpG island A was demethylated. On the contrary, the amplification of exon 2 exhibited a significant increase of mRNA levels after EPA conditioning. Interestingly, the increase of exon 2 mRNA level (Fig. 4A) was comparable to the increase of H-Ras gene expression (Fig. 2), exhibiting similar kinetics. A DNA demethylation process located upstream the start site of exon 2 and downstream exon 1 could be involved.

### EPA induces demethylation of the intron 1 CpG island B

To verify whether EPA treatment induces demethylation activity, H-Ras CpG island B (Fig. 3B), containing 26 CpG dinucleotides, was analyzed in detail (Fig. 4B). Bisulfite sequencing of this region showed high methylation levels on U937 untreated and OA treated cells (Fig. 4C). Almost complete demethylation of CpG was found in all the sequenced clones after EPA treatment (Fig. 4C). The high methylation level of CpG island B in U937 untreated cells may be responsible for the low exon 2 mRNA expression level, compared to EPA conditioning. Therefore, demethylation induced by EPA may be responsible for a change in chromatin conformation and activation of exon 2 transcription.

It is worth noticing that 165 CpG dinucleotides are present in island A (139) plus island B (26). The cytosines demethylated after EPA treatment are 21, corresponding to about 13%, a value comparable to the increase of H-Ras DNA demethylation observed using the Methyl-Profiler qPCR Primer Assay (Fig. 3D).

### EPA promotes RNA Polymerase II and p53 recruitment on intron 1 CpG island B

**Figure 5:**
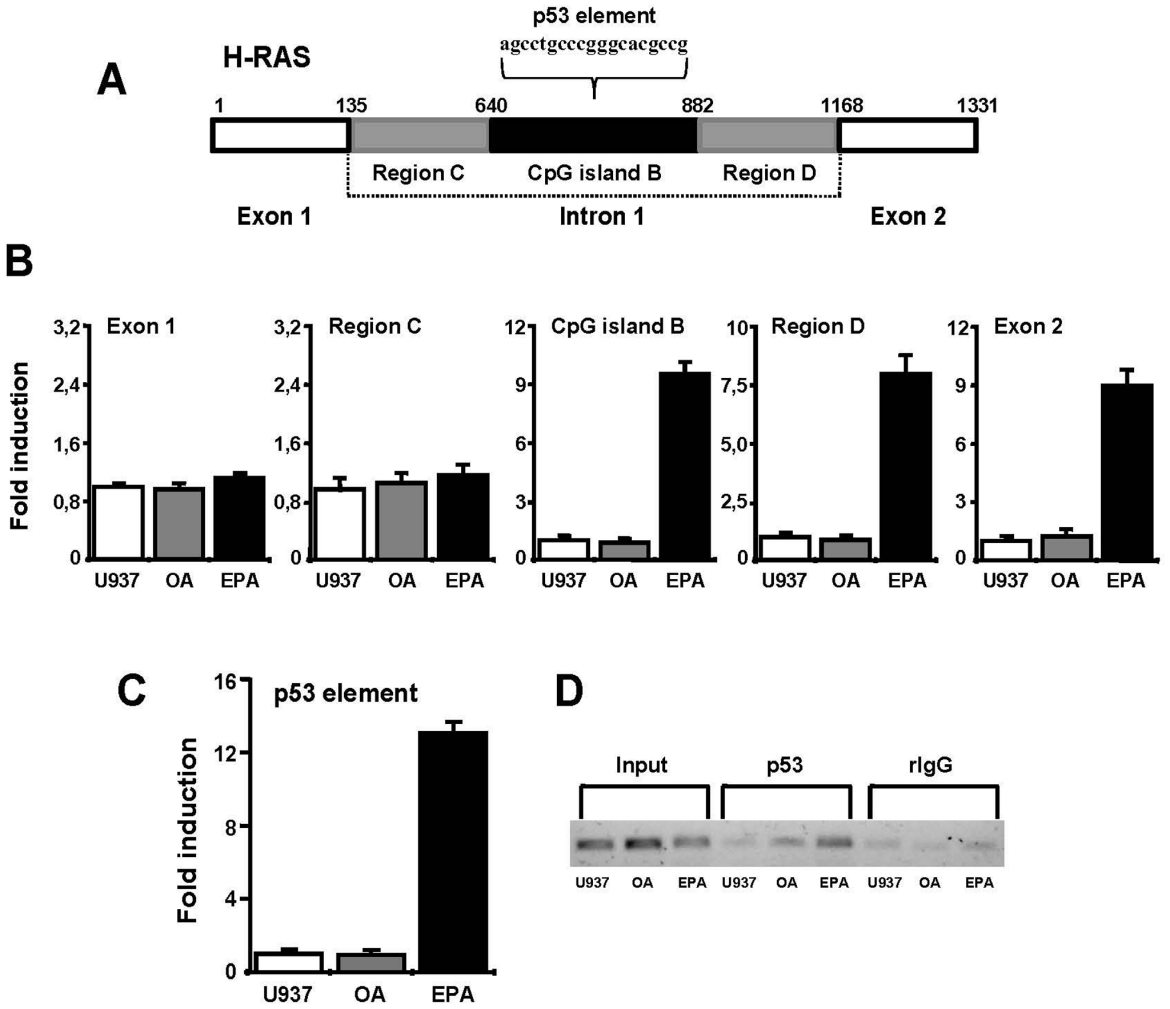
Influence of intron 1 CpG island B demethylation on RNAPII and p53 binding to H-Ras gene. (A) Schematic representation of H-Ras exon 1, intron 1, and exon 2. (B) ChIP was performed in control (*white bars*), OA (*gray bars*), and EPA treated (*black bars*) U937 cells, using RNAPII Ab. qRT-PCR was performed using specific primers for exon 1, intron 1 region C, CpG island B, intron 1 region D, and exon 2. The results shown are the mean ± SD of three independent experiments. (C) ChIP was performed as in (B) using a p53 Ab. The region within CpG island B containing the p53 element was amplified by qRT-PCR. The results shown are the mean ± SD of three independent experiments. (D) PCR of DNA from p53 Ab immunoprecipitated complex. Input, fragmented DNA before immunoprecipitation, negative controls rIgG. One representative out of three experiments is shown.

We next investigated if demethylation of intron 1 CpG island B induced by EPA is solely responsible for changes in DNA methylation pattern or the portion of CpG island A downstream from exon 1 (Fig. 3B) could be involved. For this reason we analyzed the binding of RNA Polymerase II (RNAPII) to exon 1 (1/+135), intron 1 region C (+136/+639), CpG island B (+640/+882), intron 1 region D (+883/+1167), and exon 2 (+1168/+1331) (Fig. 5A).

ChIP analysis was performed in control U937 cells and after 24 hours of OA and EPA treatments, using anti-RNAPII antibody. No differences were detected in qRT-PCR amplification of exon 1 and intron 1 region C in the studied conditions (Fig. 5B). On the contrary, a significant ability of RNAPII to bind CpG island B, intron 1 region D, and exon 2 after EPA treatment was observed (Fig. 5B). These results indicate a decreased enrichment of RNAPII on methylated CpG island B, which was removed by EPA treatment, enhancing H-Ras gene transcription.

The influence of the methylation level of CpG island B on H-Ras gene expression was further investigated by performing ChIP analysis in control U937 cells and after 24 hours of OA and EPA treatments, using an anti-p53 antibody. CpG island B contains regulatory regions, including a p53 element that functions as a transcriptional enhancer [36]. A significant increase of p53 binding to its consensus sequence on CpG island B was observed after EPA treatment, both by ChIP and PCR analysis (Fig. 5C and D). These results are concordant with the RNAPII ChIP experiments (Fig. 5B) and indicate that CpG island B DNA methylation level is essential for H-Ras gene expression.

## Discussion

In this study we show that an intronic DNA demethylation process induced by EPA is responsible for the increase of H-Ras gene and protein expression, essential for the activation of Ras/ERK/C/EBPβ pathway in U937 promonocytic cells.

The induction of C/EBPβ activity by EPA is particularly interesting, as C/EBPβ exerts a key role on proliferation and differentiation processes by regulating the expression of monocyte related genes, including C/EBPβ itself [10], [11]. Our finding that the CpG island in C/EBPβ gene is unmethylated suggests an accessible chromatin uncondensed form, allowing the binding of activated C/EBPβ that results in the increase of its transcription [10]. EPA activates C/EBPβ by promoting Thr235 phosphorylation through the activation of ERK1/2 and the increase of H-Ras protein expression levels. These results confirm previous data demonstrating that C/EBPβ becomes post-transcriptionally activated by Ras/MEK/ERK signaling during monocyte differentiation [14], [15] thus being a Ras target gene [37].

Although Ras signaling pathway is a very complex network that may control cell proliferation, survival, and differentiation [38], EPA-mediated increase of Ras proteins level appears to be involved in activating Ras/ERK/C/EBPβ pathway. This result is in accordance with the demonstration that the amplitude of Ras proteins level and activity determines the balance between proliferation and differentiation in myeloid cell fate to such an extent that a brief exposure to high H-Ras levels causes monocyte differentiation [21].

Interestingly, the increased Ras proteins levels induced by EPA and other PUFA were localized in non raft membranes, where Ras proteins activation occurs [39], [40] to promote ERK1/2 phosphorylation [32], [33]. The finding that Ras was not present in raft membranes in any of the studied conditions indicates that PUFA did not induce Ras proteins transition from rafts to other cellular membranes [35]. Indeed, the evidence that protein content and lipid composition of rafts were unchanged after PUFA conditioning, demonstrates that PUFA were unable to penetrate into rafts to induce membranes remodeling [32], [33]. Factors other than Ras transition process from raft to non raft membranes appear involved in the increase of non raft Ras proteins.

A transcriptional process is responsible for the enhancement of H-Ras and N-Ras gene and protein expression after EPA conditioning. The increase of H-Ras protein expression after both 5-aza-dC and EPA matched the increase of total Ras proteins expression, indicating that H-Ras predominates over the other isoforms and EPA exerts its effect acting as a demethylating agent.

Methylation levels of H-Ras promoter are highly related to gene expression in cancer cell lines, [41] involving a great number of regulatory elements [42], [43]. A portion of the proximal promoter region upstream exon 1 is within the CpG island A (Fig. 5 A). Since the transcription levels of exon 1 are comparable in EPA conditioned and in U937 untreated cells, we conclude that the promoter region is not involved in the enhanced expression of H-Ras induced by EPA. Similarly, the binding of RNAPII on exon 1 and intron 1 region C, both comprised within CpG island A, is comparable between control and EPA treated cells (Fig. 5 A and B), excluding changes in the RNAPII activity. As RNAPII is depleted exclusively in the DNA methylated regions [44], our finding provides the evidence that CpG island A does not exhibit changes in DNA methylation pattern after EPA conditioning and is not involved in the increase of H-Ras expression induced by EPA.

In contrast, H-Ras gene exhibits an hypermethylated intron 1 CpG island B in control U937 cells, which is almost completely demethylated after EPA conditioning, in agreement with the increase of H-Ras exon 2 transcriptional level. These data indicate a block of RNAPII recruitment and activity between the two exons, which is removed by the demethylating action of EPA, suggesting a change in intronic chromatin conformation.

It is interesting to note that elements located in H-Ras intron 1 influence its expression [36]. Indeed, Sp1, steroid hormones, and p53, able to induce H-Ras transcription levels [45]–[47], recognize sequences located on intron 1 within the CpG island B. Hypermethylation of this island may inhibit the binding of transcription factors to the recognition sequences and decrease H-Ras gene expression. We may speculate that demethylation of CpG island B induced by EPA promotes an open chromatin conformation, as demonstrated by the increase of RNAPII recruitment on CpG island B, which results in higher elongation efficiency on intron 1 region D and exon 2 (Fig. 5B). The change induced by EPA on the DNA methylation pattern is confirmed by p53 binding to its consensus sequence on CpG island B (Fig. 5C and D). The enhancement of RNAPII recruitment on CpG island B induced by EPA appears to be solely responsible for increased H-Ras transcription (Fig. 2). This result is in agreement with the fact that transcriptional regulation associated with CpG hypermethylation is not restricted to the promoter region. Actually, CpG islands may be located far downstream the transcriptional start site in introns and coding regions [48], [49]. Moreover, reduction of gene expression imputable to hypermethylation of intron 1 CpG islands has been reported in solid tumors, in leukemia cell lines, and in peripheral blasts [50], [51]. In mammalian cells, intragenic DNA methylation alters chromatin structure and RNA elongation efficiency [44], [52]. In accordance, hypermethylation of intron 1 CpG island B may modify RNAPII activity, resulting in low mRNA levels (Fig. 5A and B). As a consequence, U937 leukemia cells express H-Ras protein levels that could be too low to activate efficiently the ERK/C/EBPβ pathway. Indeed, the myeloid cell fate (proliferation *versus* differentiation) strongly depends on H-Ras protein content, high expression levels supporting the monocyte differentiation program [21].

Both 5-aza-dC and EPA conditioning induced H-Ras proteins expression. The finding that EPA is able to induce significantly H-Ras mRNA levels after 1 hour is consistent with the induced expression of the tumor suppressor gene C/EBPδ [10] and reinforces the hypothesis that active demethylation mechanism(s) occur in the absence of DNA replication. The existence of demethylating enzymes has been previously postulated when rapid demethylation of genes occurs [53]. Interestingly, among the potential mechanisms proposed for DNA demethylation, TET proteins are required to initiate the active demethylation process in differentiating monocytes [54]. In addition, TET2 expression levels are under the control of IDAX protein in U937 cells [55]. The possibility that some of these proteins are involved in the active demethylation induced by EPA in U937 cells deserves further investigation.

The effects induced by EPA on Ras/ERK/C/EBPβ pathway appear similar to those of LNA, AA, and DHA (Fig. 1). Although these PUFA belong to the n-6 and n-3 series and are precursors of bioactive molecules able to specifically regulate pathways involved in proliferation *versus* differentiation processes, a common mechanism of action cannot be excluded.

In conclusion, our findings provide the first evidence that a demethylation process induced by an endogenous fatty acid is essential for Ras/ERK/C/EBPβ pathway activation, supporting the increased expression of an early gene during the differentiation program of U937 leukemia cells. Indeed, the activation of the monocyte cell lineage specific gene M-CSF receptor needs C/EBPβ/C/EBPδ heterodimer binding to its promoter [10]. EPA plays a pivotal role as demethylating agent, by directly acting on C/EBPδ and H-Ras, an upstream C/EBPβ activator. A new role emerges for EPA and, possibly, other PUFA, which may represent a new class of DNA demethylating agents, whose action should be investigated in cancer cells, as well as in other diseases, when aberrant DNA hypermethylation is involved.
